# Dynamic Positional Changes in the Popliteal Artery and Vastus Medialis and Lateralis Muscles During Knee Flexion and Extension: An Open MRI-Based Anatomical Study

**DOI:** 10.3390/diagnostics16101455

**Published:** 2026-05-10

**Authors:** Tsubasa Hasegawa, Yuki Okazaki, Yusuke Mochizuki, Takayuki Furumatsu, Takaaki Hiranaka, Koki Kawada, Toshiki Kohara, Tomonori Tetsunaga, Toshifumi Ozaki

**Affiliations:** 1 Department of Orthopaedic Surgery, Okayama University Graduate School of Medicine, Dentistry, and Pharmaceutical Sciences, 2-5-1 Shikata-cho, Kita-ku, Okayama 700-8558, Japan; hasegawa_28377@outlook.jp (T.H.);; 2 Department of Orthopaedic Surgery, Faculty of Medicine, Okayama University, 2-5-1 Shikata-cho, Kita-ku, Okayama 700-8558, Japan; 3 Department of Orthopaedic Surgery, Okayama Red Cross Hospital, 2-1-1 Aoe, Kita-ku, Okayama 700-8607, Japan

**Keywords:** open MRI, neurovascular complications, soft tissue, popliteal artery, vastus medialis, vastus lateralis, knee surgery, osteotomy

## Abstract

**Background/Objectives**: In periarticular knee surgery, such as osteotomies, ligament reconstruction, and fracture fixation, surgeons face a dilemma: ensuring the safety of the popliteal artery (PA) while securing adequate surgical access to the bone. While macroscopic anatomical studies suggest knee flexion protects the PA, they often fail to account for physiological muscle tension in living knees. This study aimed to quantitatively evaluate the dynamic positional changes in the PA and the vastus medialis and lateralis muscles (VM and VL, respectively) using Open Magnetic Resonance Imaging (MRI) to determine the optimal limb position for each surgical step. **Methods**: Twenty-three living knees were evaluated using Open MRI. The shortest perpendicular distances from the posterior aspect of the femur and tibia to the PA, and from the femoral cortex to the posterior border of the VM and VL, were measured at 10° knee-flexed position (representing the extended position) and 90° knee-flexed position. **Results**: The PA shifted significantly away from the bone in 90° knee-flexed position compared to extension at the distal femur (0 and 1 cm proximal to the intercondylar line (Blumensaat’s line)) and the proximal tibia (0, 1, and 2 cm distal to the joint line) (*Q* < 0.05). Conversely, both the VM and VL moved significantly closer to the femur in flexion at all measured levels (0–4 cm) (*Q* < 0.05), often causing the muscles to compress tightly against the bone. **Conclusions**: The vascular safety margin is maximized in flexion, whereas surgical exposure for the distal femur is optimized in extension due to vastus muscle relaxation. We suggest performing superficial exposure and femoral plate insertion in extension, and surgical maneuvers involving the posterior cortex in flexion to minimize neurovascular and soft tissue complications.

## 1. Introduction

Various surgical procedures around the knee, such as osteotomies, ligament reconstruction, and open reduction and internal fixation (ORIF) for peri-articular fractures, require precise anatomical knowledge to avoid iatrogenic complications [1,2,3]. In particular, high tibial osteotomy (HTO) and distal femoral osteotomy (DFO) are essential for correcting limb malalignment. In these procedures, a paramount concern is the protection of the neurovascular bundle (NVB), especially the popliteal artery (PA), which courses directly posterior to the surgical field, the distal femur, and proximal tibia. Although iatrogenic injury to the PA is rare, it is a devastating complication that may result in limb-threatening ischemia or even amputation [4,5].

Minimizing vascular risk while maintaining sufficient surgical exposure, often requiring retraction of the vastus medialis (VM) or vastus lateralis (VL), is a critical aspect of periarticular surgery. However, the limb position that maximizes the vascular safety margin may not always be optimal for surgical accessibility. While a study using anatomical specimens has demonstrated that knee flexion increases the distance between the PA and the posterior tibial cortex [6], these models fail to account for the dynamic behavior of soft tissues and physiological muscle tension present in living knees. Furthermore, although minimally invasive plate osteosynthesis (MIPO) is a standard technique, increased muscle tension during knee flexion can hinder implant positioning and exposure [7,8].

Importantly, these dynamic anatomical relationships in living knees cannot be adequately assessed using conventional closed-bore magnetic resonance imaging (MRI) systems. The purpose of this study was to quantitatively evaluate the dynamic displacement of the PA and the vastus muscles relative to the femur and tibia during knee flexion and extension using an open MRI. We hypothesized that knee flexion would increase the distance to the PA, thereby enhancing the vascular safety margin, while simultaneously decreasing surgical exposure due to increased tension in the vastus muscles.

## 2. Materials and Methods

### 2.1. Study Design and Patient Selection

This retrospective cohort study was approved by our Institutional Review Board and has been performed according to the Declaration of Helsinki. All patients provided informed consent.

The study initially identified 24 patients who underwent open MRI of the knee for suspected intra-articular soft tissue pathology, such as meniscus or ligament injuries, between 1 February and 31 August 2017. This period was selected to ensure a consistent imaging protocol utilizing complete axial, coronal, and sagittal sequences required for precise multi-planar reconstruction (MPR).

Inclusion criteria required the availability of valid MPR datasets in both extension and flexion positions. One patient was excluded due to severe tortuosity of the PA, which precluded consistent identification of the reference landmarks and reliable linear measurements. No other specific clinical exclusion criteria were applied, provided that knee anatomy was sufficiently preserved. Consequently, 23 patients were included in the final analysis.

An a priori power analysis was performed using pilot data from the first 5 patients. The displacement of the PA, representing the anterior limit of the NVB, at 1 cm distal to the tibial plateau was selected as the reference variable. This parameter was chosen because it demonstrated a smaller effect size (Cohen’s dz = 0.72) compared to the femoral level or vastus muscles, representing a conservative estimate. The analysis indicated that a minimum of 18 knees was required to detect a significant difference (power: 80%, alpha: 0.05). Since the final sample size (*n* = 23) exceeded this requirement, the study was deemed to have sufficient statistical power.

### 2.2. MRI Acquisition Protocol

Imaging was performed using a 1.2-T open MRI system (OASIS, Hitachi Medical Corp., Chiba, Japan). Unlike conventional MRI scanners, this open-gantry system allows image acquisition under surgically relevant positions, including deep flexion, while preserving physiological muscle tension. Each knee was imaged in two standardized limb positions designed to simulate key phases of periarticular knee surgery. To ensure reproducibility, the hip was maintained in neutral rotation and the tibia was positioned without internal or external rotation. For 10° knee-flexed position, patients were placed in the supine position with the patella facing upward and the second toe oriented vertically. For 90° knee-flexed position, patients were placed in the lateral decubitus position with the affected side down due to gantry constraints while maintaining neutral alignment.

1. 10° knee-flexed position: When placed in the supine position on the scanner table, the knee naturally rests at approximately 10° of knee flexion rather than at a forced 0° of full extension. Consequently, this relaxed posture accurately replicates the clinical “extension” position typically maintained using a heel support during surgery, which corresponds to the surgical phase involving skin incision, superficial dissection, fluoroscopic alignment, and medial meniscus procedures.
2. 90° knee-flexed position: The knee was flexed to approximately 90°, corresponding to surgical phases that require posterior access, including posterior cortical drilling, completion of osteotomy of the posterior cortex, and lateral meniscus procedures.

Proton density-weighted images were primarily acquired in the sagittal and coronal planes to optimize soft tissue contrast, while axial images were obtained using T2-weighted sequences to enhance visualization of neurovascular structures and to serve as a reference indicator for the coronal and sagittal planes. For quantitative analysis, sagittal MPR images were generated perpendicular to the posterior condylar axis to ensure consistent and reproducible measurement planes across subjects and positions.

### 2.3. Measurements and Landmarks

All measurements were performed on sagittal MPR images using a digital image analysis workstation (Picture Archiving and Communication System). Two independent observers performed the measurements. Distances were measured at 1-cm intervals from predefined anatomical reference points to evaluate position-dependent changes in the vascular safety margin and surgical exposure. The mean value of the measurements obtained by the two observers was used for the final statistical analysis. To ensure measurement consistency across different anatomical planes, the reference lines for the posterior bone cortices were established on the digital workstation and maintained as a fixed overlay while scrolling through the sagittal slices. This technique allowed for the precise measurement of distances to the PA, VM, and VL on their respective optimal sagittal slices relative to the same established bone reference.

**Figure 1:**
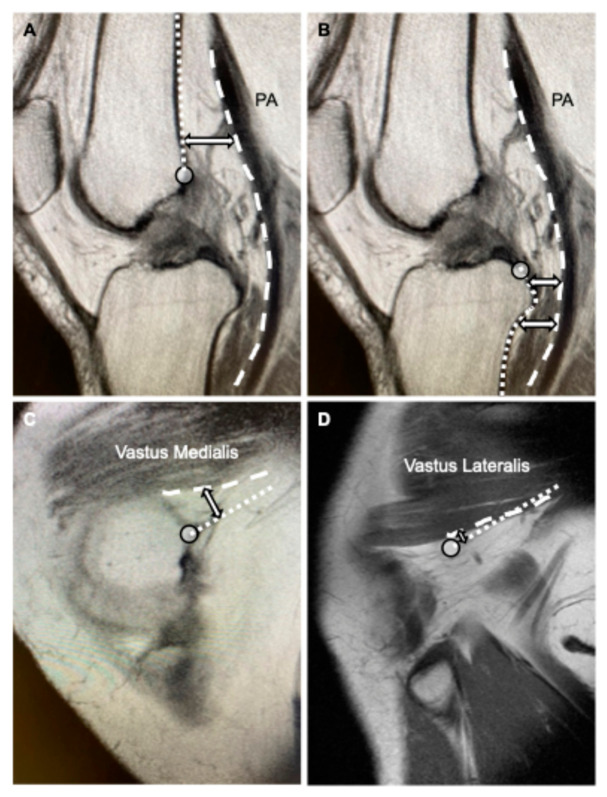
Measurement methods defined on open magnetic resonance imaging (MRI) images. (**A**) Measurement of the vascular safety margin on the femoral side (F-PA): On the sagittal multi-planar reconstruction (MPR) image, the distance from the posterior cortex of the femur (dotted line) to the anterior wall of the popliteal artery (PA) (dashed line) was measured perpendicular to the longitudinal axis of the femur. The circle marker indicates the zero reference point (0 cm) at the intersection of the intercondylar line (Blumensaat’s line) and the posterior femoral cortex. (**B**) Measurement of the vascular safety margin on the tibial side (T-PA): On the sagittal MPR image, the distance from the posterior cortex of the tibia (dotted line) to the anterior wall of the PA (dashed line) was measured perpendicular to the longitudinal axis of the tibia. The circle marker indicates the zero reference point (0 cm) at the intersection of the tibial plateau (joint line) and the posterior tibial cortex. (**C**) Measurement of surgical exposure for the vastus medialis (F-VM): On the medial sagittal MPR image, the distance from the posteromedial femoral cortex (dotted line) to the posterior border of the vastus medialis (F-VM) (dashed line) was measured perpendicular to the longitudinal axis of the femur to evaluate the potential space available for muscle retraction. The circle marker indicates the zero reference point (0 cm) at the intersection of the intercondylar line and the posteromedial femoral cortex. (**D**) Measurement of surgical exposure for the vastus lateralis (F-VL): On the lateral sagittal MPR image, the distance from the posterolateral femoral cortex (dotted line) to the posterior border of the vastus lateralis (F-VL) (dashed line) was measured similarly. The circle marker indicates the zero reference point (0 cm) at the intersection of the intercondylar line and the posterolateral femoral cortex. The double-headed arrows in each panel indicate the measurement direction, perpendicular to the longitudinal axis of the respective bone.

• Reference Points:

1. Femoral side: The intersection of the intercondylar line (Blumensaat’s line) with the posterior femoral cortex was defined as the zero reference point (0 cm). Measurements were obtained at 0, 1, 2, 3, and 4 cm proximal to this point (Figure 1A). The intercondylar line was selected as the reference axis because it is a universally recognized radiographic landmark for determining the anatomical femoral attachment and tunnel placement in anterior cruciate ligament reconstruction and preservation surgeries [9,10].
2. Tibial side: The posterior aspect of the tibial plateau (joint line) was defined as the zero reference point (0 cm). Measurements were obtained at 0, 1, 2, 3, and 4 cm distal to this point (Figure 1B). The joint line was chosen as the primary baseline because it is the essential landmark for determining the osteotomy hinge position and safe screw placement levels in periarticular tibial surgery [11].

• Evaluated Distances:

1. Vascular Safety Margin (F-PA and T-PA): The distance from the posterior cortex of the femur (F) or tibia (T) to the anterior wall of the PA was measured perpendicular to the longitudinal axis of the respective bone (Figure 1A,B). As the popliteal vein and tibial nerve are typically located posterior to the artery, the PA was used as a surrogate marker representing the closest component of NVB to the bone.
2. Surgical Exposure (F-VM and F-VL): To evaluate the potential space available for muscle retraction or implant insertion, the distance from the posteromedial femoral cortex to the posterior border of the vastus medialis (VM) (F-VM) (Figure 1C) and from the posterolateral femoral cortex to the posterior border of the vastus lateralis (VL) (F-VL) (Figure 1D) was measured perpendicular to the longitudinal axis of the femur at the predefined femoral levels.

A positive value indicates the presence of a free space between the muscle and the bone, whereas a negative value indicates that the muscle was in direct contact with or had wrapped anteriorly around the bone, suggesting that no potential space was available without forceful retraction.

### 2.4. Statistical Analysis

Continuous variables are presented as mean ± standard deviation (SD). To assess the reliability of the measurements, the intraclass correlation coefficients (ICC) were calculated. Inter-observer reliability was evaluated by comparing the measurements obtained by the two independent observers for all patients. Intra-observer reliability was assessed by having one observer repeat the measurements for a subset of randomly selected knees (*n* = 10) after an interval of at least two weeks. The ICC value > 0.80 was defined as indicating excellent reliability. Differences in measurements between 10° knee-flexed position and 90° knee-flexed position were analyzed using paired *t*-tests, as all measurements were obtained from the same knee under two different limb positions. The normality of the data distribution was confirmed. To account for multiple comparisons across the five measurement levels (0, 1, 2, 3, and 4 cm), the false discovery rate (FDR) was controlled using the two-stage step-up method of Benjamini, Krieger, and Yekutieli, with the desired FDR (*Q*) set to 5%. This approach was selected to balance the risk of type I and type II errors in this exploratory anatomical imaging study. All statistical analyses were performed using GraphPad Prism version 10 (GraphPad Software, Boston, MA, USA). Statistical significance was defined as an FDR-adjusted *Q*-value < 0.05.

## 3. Results

The inter-observer and intra-observer reliability for all MRI measurements were excellent, with the ICC values ranging from 0.82 to 0.89.

### 3.1. Patient Demographics

The study included 23 patients (23 knees) with a mean age of 43.3 ± 19.3 years (range: 17–77 years). The cohort consisted of 10 males (43.5%) and 13 females (56.5%). The primary diagnoses included medial meniscus injury (65.2%), anterior cruciate ligament rupture (47.8%), and lateral meniscus injury (30.4%) (Table 1).

### 3.2. Vascular Safety Margin (PA)

**Figure 2:**
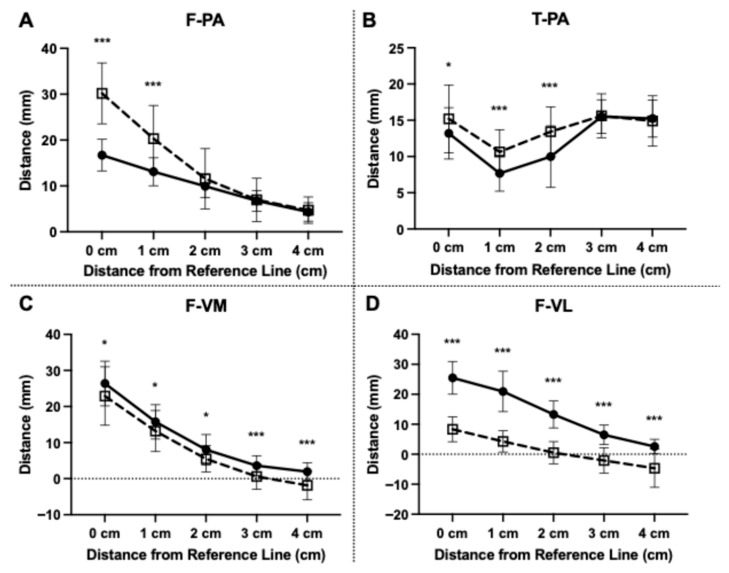
Quantitative analysis of the distances from the posterior cortex of the femur and tibia to the popliteal artery (PA) and the vastus medialis and lateralis muscles. Data are presented as mean ± standard deviation. The solid line with filled circles represents 10° knee-flexed position, and the dashed line with open squares represents 90° knee-flexed position. (**A**) Femoral side (F-PA): The distance to the PA significantly increased in the flexion position at 0 and 1 cm proximal to the intercondylar line (Blumensaat’s line) compared with the extension position. (**B**) Tibial side (T-PA): The safety margin significantly increased in the flexion position at 0–2 cm distal to the tibial plateau (joint line) compared with the extension position. (**C**,**D**) Vastus medialis and lateralis muscles (F-VM and F-VL): The distance to the vastus medialis (**C**) and vastus lateralis (**D**) significantly decreased in the flexion position compared with the extension position across all levels, indicating increased muscle tightness. The dotted line at 0 mm represents the position of the femoral bone cortex; negative values indicate that the muscle wraps anteriorly around the femur, eliminating the potential space for retraction. Bars represent standard deviations. Asterisks indicate statistical significance as determined by paired *t*-test. *: *Q* < 0.05, ***: *Q* < 0.001.

Quantitative analysis demonstrated that knee flexion significantly increased the distance between the popliteal artery and the posterior aspect of the bone, particularly near the joint line (Figure 2A,B) (Table 2). On the femoral side, at the level of 0 cm (the intercondylar line), the F-PA increased significantly from a mean of 16.7 ± 3.5 mm in extension to 30.2 ± 6.6 mm in flexion (*Q* < 0.001). This displacement represents a safety margin expansion of approximately 13.5 mm at the distal femur. A significant increase was also observed at 1 cm proximal to the reference line. However, at the more proximal levels (2, 3, and 4 cm), no significant differences were observed between the two positions. At the 4 cm level, the artery remained in close proximity to the bone (4.3 ± 2.0 mm in extension and 4.7 ± 2.9 mm in flexion) in both extension and flexion.

**Figure 3:**
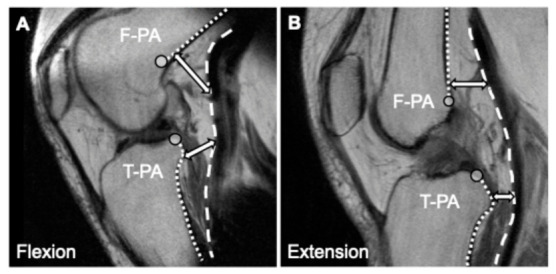
Representative sagittal magnetic resonance imaging (MRI) demonstrating the dynamic positional changes in the popliteal artery (PA) between the flexion and extension positions. (**A**) 90° knee-flexed position and (**B**) 10° knee-flexed position. The double-headed arrows indicate the measurement direction, representing the distance from the posterior bone cortices (dotted lines) to the PA (dashed line), measured perpendicular to the longitudinal axis of the respective bone. As visually demonstrated in these representative images, the quantitative analysis confirmed that the vascular safety margin significantly increased in the flexion position at 0 and 1 cm proximal to the intercondylar line (Blumensaat’s line) on the femoral side (F-PA), and at 0–2 cm distal to the tibial plateau (joint line) on the tibial side (T-PA). The circle markers indicate the zero reference points (0 cm), located at the intersections of the anatomical reference lines (the intercondylar line and the tibial plateau) and the respective posterior bone cortices.

On the tibial side, the T-PA significantly increased in flexion at 0 (joint line), 1, and 2 cm distal to the joint line (*Q* < 0.05). At the 2 cm level, the distance increased by approximately 3.5 mm in flexion compared to extension. No significant differences were found at the 3 cm and 4 cm distal levels. Representative MRIs visually corroborating this dynamic posterior displacement and the expansion of the vascular safety margin are shown in Figure 3.

### 3.3. Topographic Relation Between the Femur and the VM and VL in Extension vs. Flexion

In contrast to the vascular structures, the vastus muscles moved significantly closer to the femur during flexion (Figure 2C,D) (Table 3). For the VM, the F-VM decreased significantly in flexion at all measured levels (0, 1, 2, 3, and 4 cm) proximal to the reference line. Notably, at the 4 cm proximal level, the mean distance decreased from 1.9 ± 2.5 mm in extension to −1.9 ± 4.0 mm in flexion. This negative value indicates that the muscle wrapped anteriorly around the bone.

**Figure 4:**
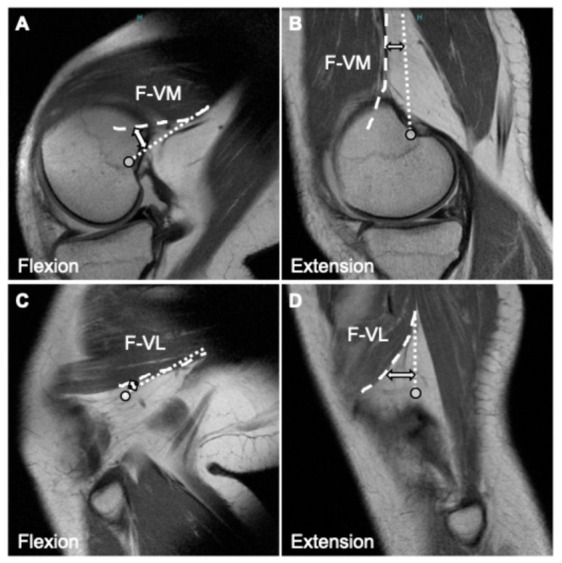
Representative sagittal magnetic resonance imaging (MRI) demonstrating the dynamic positional changes in the vastus medialis (VM) and vastus lateralis (VL) relative to the femur between 10° and 90° knee-flexed positions. (**A**,**B**) The distance from the posteromedial femoral cortex to the posterior border of the VM (F-VM): A representative example in the flexion (**A**) and extension (**B**) positions. (**C**,**D**) The distance from the posteromedial femoral cortex to the posterior border of the VL (F-VL): A representative example in the flexion (**C**) and extension (**D**) positions. The double-headed arrows indicate the measurement direction, representing the shortest perpendicular distance from the femoral bone cortex (dotted lines; posteromedial in (**A**,**B**) and posterolateral in (**C**,**D**)) to the posterior border of the respective muscles (dashed line; VM in (**A**,**B**) and VL in (**C**,**D**)), measured perpendicular to the longitudinal axis of the femur. As visually demonstrated in these representative images, the quantitative analysis confirmed that the F-VM and F-VL were significantly greater in the extension position compared to the flexion position across all measured levels (0–4 cm). The circle markers represent the predefined reference points (0 cm) consistent with the definitions in Figure 1. The circle markers indicate the zero reference points (0 cm) located at the intersections of the intercondylar line (Blumensaat’s line) and the respective posterior femoral cortices.

Similarly, the F-VL was significantly shorter in flexion across all measured levels (0–4 cm) compared to extension (*Q* < 0.05). Notably, at the 4 cm level, the distance decreased drastically from 2.6 ± 2.4 mm in extension to −4.7 ± 6.4 mm in flexion. Representative MRIs visually corroborating the decreased muscle distances during flexion are shown in Figure 4.

## 4. Discussion

This study elucidates the conflicting anatomical behaviors of NVB and muscular envelope around the knee under different limb positions in living patients. These findings have direct implications for operative techniques in knee osteotomies, reconstructive surgery, and orthopedic trauma. Our quantitative analysis demonstrated a critical trade-off: knee flexion enhances the vascular safety margin by increasing the distance between the bone and the PA, whereas surgical exposure is compromised due to increased tension within the extensor mechanism.

The primary finding regarding the vascular safety margin can be explained by the anatomical tethering of the PA. The artery is anchored proximally at the adductor hiatus and distally at the soleal arch [12]. In knee extension, the vessel is stretched and pressed against the posterior capsule. In contrast, knee flexion shortens the distance between these tethering points, introducing slack that allows the artery to drift posteriorly away from the bone [6]. Our data demonstrated that this effect was most pronounced around the distal femur and proximal tibia, extending to the joint line level.

Importantly, a distinction must be made between femoral and tibial regions. On the femoral side, particularly at the more proximal levels (2–4 cm proximal to the joint line), the distance to the PA and the bone did not significantly increase even in flexion. This suggests that the proximal tethering effect at the adductor hiatus remains dominant in this region. Clinically, this finding is critical: knee flexion does not guarantee the vascular safety margin during proximal screw insertion for distal femoral plates, and caution is still required despite flexion. In contrast, on the tibial side, the expansion of the vascular safety margin peaked at 2 cm distal to the joint line. This level corresponds precisely to the common zone for proximal tibial screws or osteotomy cuts, including the standard hinge point used in MOWHTO [4,13]. Thus, our study provides in vivo anatomical evidence that knee flexion is the most effective strategy for protecting the popliteal artery in this specific “danger zone”, where the risk of injury from osteotomes or drills is highest [6,14]. Although an absolute expansion of 2.0 to 3.5 mm may appear numerically modest, in the confined posterior compartment, this clearance provides the exact margin of error required to accommodate inadvertent drill over-penetration or to safely place a protective retractor. Accordingly, during osteotomies such as HTO or DFO, surgical maneuvers involving the posterior cortex, recognized as a region of increased risk to the PA [12], are optimally performed with the knee in flexion. A similar principle applies to trauma and reconstruction procedures: when drilling or placing screws across the posterior cortex, such as in distal femoral fractures or tibial plateau fracture fixation, maintaining knee flexion may reduce the risk of NVB injury in cases of inadvertent posterior cortical penetration. In recent clinical practice, intraoperative ultrasound is increasingly utilized as a useful adjunct for real-time identification of vascular structures. However, its use remains operator-dependent and is not routinely implemented in all surgical settings. Therefore, a fundamental understanding of these dynamic anatomical changes provides surgeons with a foundational, equipment-free knowledge base to enhance safety and operative confidence.

These results corroborate previous anatomical specimen findings [1,6,15], including angiographic evaluations by Kim et al., three-dimensional CT analysis by Bisicchia et al., and a recent anatomical study by Mori et al., as well as MRI observations in living knees by Shiomi et al. [16]. However, anatomical specimen studies inherently lack active muscle tone and fluid dynamics. Furthermore, they often require extensive dissection, such as sectioning the posterior cruciate ligament and posterior capsule, or aggressive muscle detachment and chemical soft-tissue debridement, to visualize the vessels directly. These invasive preparations inevitably alter the natural knee kinematics, tissue compliance, and structural integrity. By evaluating living knees, our study overcomes these limitations, confirming that the vascular safety margin generally increases with knee flexion under completely intact anatomical conditions. Furthermore, while a previous study in living knees was limited solely to the tibial side [16], our study significantly expands upon this foundation by systematically mapping both the femoral and tibial sides. Most importantly, we simultaneously quantified the opposing dynamic changes in the vastus muscles. This comprehensive approach demonstrates not just the expansion of the vascular safety margin, but the critical anatomical trade-off between vascular protection and surgical exposure. However, knee flexion does not completely eliminate the risk of NVB injury. In some cases, the PA remains close to the posterior cortex even in flexion, potentially due to individual anatomical variations or the mass effect of surrounding muscles such as the popliteus muscle [12]. This underscores the importance of individualized preoperative assessment using imaging modalities. Conversely, our analysis of muscle accessibility revealed that the quadriceps mechanism tightens and compresses against the femur during knee flexion. Although MIPO was originally developed to preserve the biological environment by minimizing soft tissue stripping [7], excessive muscular tension can hinder implant maneuverability. Such mechanical constraints have been associated with malalignment, technical difficulty, and an increased risk of iatrogenic injury due to limited visualization [17].

This study quantitatively substantiates these clinical observations. The significant reduction in muscle-to-bone distance observed during flexion, often reaching negative values, effectively eliminates the potential space required for plate insertion and creates a mechanical barrier that may obstruct the lateral surgical approach. This explains why deep manipulation or plate tracking in this position risks muscle stripping or unintended soft tissue injury. In contrast, knee extension allows the relaxation of the quadriceps muscles, creating a natural potential space that facilitates atraumatic exposure and implant positioning [18].

Based on these position-dependent discrepancies, optimizing limb positioning according to the specific surgical step, rather than performing the entire procedure in a single static position, may be a beneficial strategy. The 10° knee flexed position is potentially the optimal position for superficial dissection, muscle retraction, and plate insertion, whereas the 90° knee flexed position is optimal for maneuvers involving the posterior cortex, such as drilling, screw insertion, or completion of osteotomy. This stepwise adjustment in limb position allows surgeons to exploit the advantages of both anatomical states and may reduce complications during procedures such as MOWHTO, DFO, and periarticular fracture fixation.

There are some limitations in this study. First, the study population consisted of patients with knee pathology rather than healthy volunteers; however, these demographics reflect a patient population typical for knee joint preservation surgery or ligament reconstruction [19,20]. Second, only two static positions were evaluated (10° and 90°), and the behavior of soft tissue at intermediate flexion angles remains unknown. Furthermore, the 10° knee flexed position does not represent true full extension. Our findings should therefore be interpreted strictly as a comparison between 10° and 90° of knee flexion rather than absolute extension and flexion states. Third, although Open MRI enabled imaging under surgically relevant positions, its 1.2-T magnetic field offers lower spatial resolution compared to contemporary 3.0-T closed-bore systems. However, modern 1.2-T scanners are classified as high-field open MRIs and have been reported to provide excellent image quality sufficient for evaluating macroscopic musculoskeletal structures [21]. All images were obtained under non-weight-bearing conditions. Specifically, the 90° knee flexed position images were acquired in the lateral decubitus position due to gantry constraints; this differs from the supine position (used for the 10° knee flexed position images) typically used during surgery. This positional change introduces a potential confounding factor, as gravitational forces and soft tissue shifts may influence the observed measurements. Finally, the PA was used as a surrogate for the NVB; although the tibial nerve generally lies more posteriorly, individual anatomical variations exist. Furthermore, smaller branching vessels such as the genicular arteries were not evaluated. While genicular artery injury can cause postoperative hematoma or pseudoaneurysm [6,22], our primary focus was strictly on the PA, as its injury represents a catastrophic complication that can lead to limb-threatening ischemia or amputation.

## 5. Conclusions

This MRI study of living knees demonstrates a critical trade-off between the vascular safety margin and surgical exposure governed by knee flexion angle. Knee flexion displaces the PA away from the bone, maximizing the vascular safety margin, whereas knee extension allows the relaxation of the vastus muscles and improves surgical exposure. While recognizing the limitations of static, non-weight-bearing imaging, our findings suggest that surgeons performing periarticular knee surgeries, including osteotomy, reconstruction, and trauma fixation, might optimize limb positioning according to the specific surgical step. These anatomical observations support the hypothesis that utilizing knee extension for exposure and implant insertion and knee flexion for posterior cortical instrumentation enhances surgical precision and minimizes the risk of neurovascular complications.

## Figures and Tables

**Table 1:** Patient Demographics.

Characteristic	Value
Age (years)	
Mean ± Standard deviation	43.3 ± 19.3
Range	17–77
Sex, *n* (%)	
Male	10 (43.5%)
Female	13 (56.5%)
Primary Diagnosis, *n* (%)	
Medial Meniscus Injury	15 (65.2%)
Anterior Cruciate Ligament Injury	11 (47.8%)
Lateral Meniscus Injury	7 (30.4%)
Spontaneous Osteonecrosis of the Knee	1 (4.3%)

**Table 2:** Comparison of the distance between the popliteal artery and the posterior cortex of the femur (F-PA) and tibia (T-PA) between extension and flexion positions.

Measurement Level	Extension (10°) (mm)	Flexion (90°) (mm)	Mean Difference (mm)	*Q*-Value
**F-PA**				
**0 cm (the intercondylar line)**	16.7 ± 3.5	30.2 ± 6.6	+13.5	<0.001 *
**1 cm proximal**	13.1 ± 3.1	20.3 ± 7.3	+7.2	<0.001 *
**2 cm proximal**	9.9 ± 2.5	11.6 ± 6.6	+1.6	0.176
**3 cm proximal**	6.8 ± 2.3	7.0 ± 4.7	+0.2	0.823
**4 cm proximal**	4.3 ± 2.0	4.7 ± 2.9	+0.4	0.422
**T-PA**				
**0 cm (joint line)**	13.2 ± 3.6	15.2 ± 4.7	+2.0	0.037 *
**1 cm distal**	7.7 ± 2.5	10.6 ± 3.1	+3.0	<0.001 *
**2 cm distal**	10.0 ± 4.3	13.4 ± 3.4	+3.5	<0.001 *
**3 cm distal**	15.5 ± 2.3	15.6 ± 3.0	+0.1	0.831
**4 cm distal**	15.2 ± 2.6	14.9 ± 3.5	−0.3	0.497

Values are presented as mean ± standard deviation. Mean Difference is calculated as (Flexion—Extension). Positive values indicate an increase in distance during flexion. * Significant differences were defined as an FDR-adjusted *Q*-value < 0.05 following paired *t*-tests.

**Table 3:** Comparison of the distance from the femoral cortex to the vastus medialis (F-VM) and vastus lateralis (F-VL) between extension and flexion positions.

Measurement Level	10° Knee-Flexed Position (mm)	90° Knee-Flexed Position (mm)	Mean Difference (mm)	*Q*-Value
**F-VM**				
**0 cm (the intercondylar line)**	26.4 ± 6.2	22.9 ± 8.1	−3.5 ± 7.6	0.039 *
**1 cm proximal**	15.8 ± 4.8	13.2 ± 5.7	−2.6 ± 5.5	0.037 *
**2 cm proximal**	8.0 ± 4.2	5.5 ± 3.6	−2.5 ± 4.5	0.013 *
**3 cm proximal**	3.6 ± 2.8	0.6 ± 3.5	−3.0 ± 3.8	<0.001 *
**4 cm proximal**	1.9 ± 2.5	−1.9 ± 4.0	−3.8 ± 4.3	<0.001 *
**F-VL**				
**0 cm (the intercondylar line)**	25.5 ± 5.5	8.3 ± 4.1	−17.2 ± 5.4	<0.001 *
**1 cm proximal**	21.0 ± 6.7	4.3 ± 3.5	−16.7 ± 6.0	<0.001 *
**2 cm proximal**	13.3 ± 4.5	0.5 ± 3.7	−12.8 ± 5.1	<0.001 *
**3 cm proximal**	6.5 ± 3.3	−2.1 ± 4.2	−8.6 ± 4.5	<0.001 *
**4 cm proximal**	2.6 ± 2.4	−4.7 ± 6.4	−7.3 ± 6.7	<0.001 *

Values are presented as mean ± standard deviation. Mean Difference is calculated as (Flexion—Extension). Positive values indicate an increase in distance during flexion. * Significant differences were defined as an FDR-adjusted *Q*-value < 0.05 following paired *t*-tests.

## Data Availability

The data presented in this study are available on request from the corresponding author due to privacy restrictions.

## Abbreviations

The following abbreviations are used in this manuscript:

MRI	Magnetic Resonance Imaging
PA	Popliteal Artery
NVB	Neurovascular Bundle
VM	Vastus Medialis
VL	Vastus Lateralis
HTO	High Tibial Osteotomy
MOWHTO	Medial Opening Wedge High Tibial Osteotomy
DFO	Distal Femoral Osteotomy
ORIF	Open Reduction and Internal Fixation
MIPO	Minimally Invasive Plate Osteosynthesis
MPR	Multi-Planar Reconstruction
FDR	False Discovery Rate
